# GATA4/6 regulate DHH transcription in rat adrenocortical autografts

**DOI:** 10.1038/s41598-019-57351-5

**Published:** 2020-01-16

**Authors:** Takashi Yoshida, Nae Takizawa, Tadashi Matsuda, Hisao Yamada, Masaaki Kitada, Susumu Tanaka

**Affiliations:** 1 Department of Anatomy, Kansai Medical University, Hirakata, Osaka 573-1010 Japan; 2 Department of Urology and Andrology, Kansai Medical University, Hirakata, Osaka 573-1010 Japan

**Keywords:** Genomic analysis, Mechanisms of disease

## Abstract

Adrenal cortex autotransplantation with ACTH stimulation may be an alternative therapy for patients with bilateral adrenalectomy to avoid adrenal crisis, but its underlying mechanism has not been elucidated. Previously, we detected *Dhh* upregulation in rat adrenocortical autografts after transplantation. Here, we investigated potential regulators such as *Gata4*, *Gata6*, *Sry* and *Sox9* which affect *Dhh* transcription in adrenocortical autografts with or without ACTH stimulation. In ACTH-stimulated autografts, *Gata4* and *Gata6* were downregulated compared to control autografts. This response was linked to *rDhh* repression. A reporter assay using the upstream region of *rDhh* and a GATA binding motif revealed that *rDhh* promoters were significantly upregulated by co-transfection with *Gata4* or *Gata6* or both. *Sry* and *Sox9* expression in autografts with or without ACTH stimulation were verified by PCR and RNAscope analyses. The ovarian differentiation factors *Foxl2* and *Rspo1* were also upregulated in the autografts. *Gata4* and *Gata6* were found to be significant factors in the regulation of *rDhh* expression and could be associated with adrenocortical autograft maintenance. Gonadal primordia with bipotential testicular and ovarian functions may also be present in these autografts.

## Introduction

Pheochromocytomas arise from the adrenal medulla and are catecholamine-producing tumours. Hereditary pheochromocytoma can be treated with bilateral adrenalectomy and lifelong glucocorticoid replacement therapy^1^. Autotransplantation and allotransplantation of the adrenal cortex are potential alternatives that allow bilateral adrenalectomy patients to avoid adrenal crises^1,2^. However, adrenal autotransplantation has not been established in humans and its success rate is only 20–35%^3,4^. Possible reasons for this poor performance include ACTH suppression by negative feedback from excessive postoperative glucocorticoid replacement therapy. This response causes autograft regression. According to previous reports, adrenal autotransplantation has been highly successful in the management of Cushing’s disease (ACTH hypersecretion from the pars distalis)^5–7^. Four patients who underwent bilateral adrenalectomy and ACTH replacement were able to withdraw from glucocorticoid replacement immediately after adrenal autotransplantation^8^. Dexamethasone-induced adrenal atrophy in mice was restored with daily ACTH stimulation^9^. ACTH stimulation after autotransplantation preserves autografts and may involve an unidentified pathway which promotes adrenal cortical regeneration and recovers endocrine function.

In the search for factors affecting post-transplant adrenocortical autograft remodelling and regeneration, we found that *Dhh* was upregulated and *Shh* was downregulated in the regeneration step of rat adrenocortical autograft^8^. The *HH* signalling pathway may participate in adrenocortical autograft regeneration as well as adrenal cortex development. The regulation of *Dhh* transcription during gonadal development involves transcription factors such as *Wt1*, *Gata4*, *Gata6*, *Sox9* and *Sry*^9,10^. In this study, we examined whether they affect *Dhh* transcription in adrenocortical autografts. Although ACTH stimulation is important, to the best of our knowledge, no studies have evaluated the influence of transient ACTH stimulation on adrenal autografts. Therefore, we also assessed the effects of transient ACTH and *rDhh* transcription-associated factors.

## Results and Discussion

### ACTH stimulation induced angiogenic factor in adrenal glands

**Figure 1:**
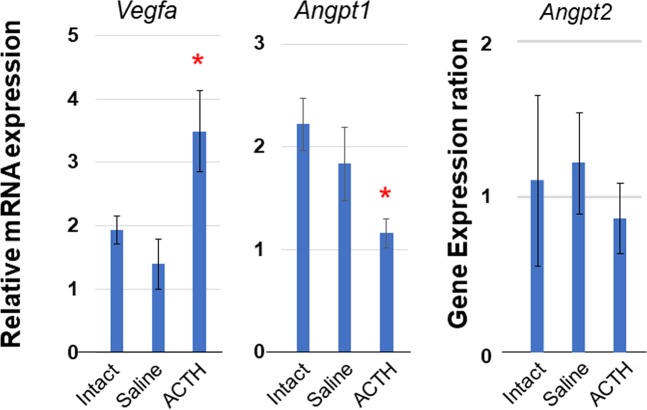
Relative expression of angiogenesis factors in adrenal glands. Rats without injection (Intact group), injected with natural saline (Saline group) or injected with ACTH (ACTH group) were euthanized after 2 h. Relative expression levels in the adrenal glands were evaluated by RT-qPCR. Changes in transcription level were analysed by ANOVA with a Steel multiple comparisons test ^*^P < 0.05 vs. Intact group. Vegfa: vascular endothelial growth factor a; Angpt: Angiopoietin.

After ACTH stimulation for 2 h, *Vegfa* showed a 1.8-fold increase in the adrenal gland compared with that in the intact group (Fig. 1). *Angpt1* in the adrenal gland after ACTH stimulation showed a 0.5-fold decrease compared with that in the intact group (Fig. 1). There was no difference between the intact and saline groups in terms of *Vegfa* and *Angpt1* expression (Fig. 1). Further, no differences in *Angpt2* expression among the three groups were observed (Fig. 1). These results corroborated those of previous reports^11–13^. Even the 2-h ACTH stimulation of the adrenal gland in the present study suggested that ACTH regulates angiogenic factors which could affect adrenal autograft conditions.

### ACTH suppressed the HH signal in the adrenal autografts

**Figure 2:**
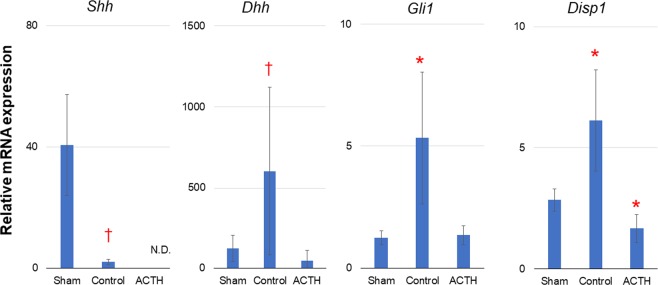
Relative expression of HH signalling molecules in adrenal tissues. Rats with Sham operation (Sham), adrenocortical autotransplantation (Control group) or adrenocortical autotransplantation plus ACTH (ACTH group) were sacrificed at POD14. Relative expression levels in the adrenal tissues were evaluated by RT-qPCR. Changes in transcription level were analysed by ANOVA with a Steel multiple comparisons test; ^*^P < 0.05 vs. Sham rats. For non-parametric factors such as *Shh* and *Dhh*, the Mann-Whitney *U* test with a Bonferroni correction was used; ^†^*P* < 0.0167 vs. Sham rats. Kif7: kinesin family member 7; Ptch1: human patched-1; Shh: sonic hedgehog; Smo: Smoothened; Sufu: Sufu negative regulator of hedgehog signalling; Dhh: desert hedgehog; Gli1: GLI family zinc finger 1; Disp1: Dispatched RND Transporter Family Member 1.

RNAscope analysis in our previous study confirmed that *Shh* was downregulated and *Dhh* was upregulated in the autografts 2–3 wks after surgery^10^. Similarly, *Shh* expression showed a 0.05-fold decrease in the control autografts compared to that in the sham (Fig. 2). *Dhh* expression was 5-fold higher in the autografts than in the sham (Fig. 2). In the ACTH-stimulated autograft, neither *Shh* nor *Dhh* was upregulated in the adrenocortical autografts relative to the sham. Similar results were observed for *Gil1* expression (4.2-fold increase in the control autografts, no difference in the ACTH-stimulated autograft) (Fig. 2). Therefore, 2-h ACTH stimulation dysregulated HH signal-related genes in the autografts 2 wks after surgery.

*Disp1* encodes HH ligand secretion receptors and is co-localised with the HH ligand in the same cells^14^. *Disp1* showed a 2.1-fold upregulation in the control autograft but a 0.6-fold downregulation by ACTH stimulation (Fig. 2). Therefore, both DHH synthesis and release were suppressed in the DHH-producing cells of the ACTH-stimulated autograft. On postoperative day (POD)14, there might be slight HH ligand binding in the autograft HH target cells.

### Transcriptional Dhh regulator and effect of ACTH stimulation

The expression of certain *Dhh* transcriptional regulators may be linked to *Dhh* expression in the 2–3 wks after surgery, during which time *Dhh* was upregulated. Therefore, we measured the expression levels of candidate transcription factors in adrenal autografts at POD14.

**Figure 3:**
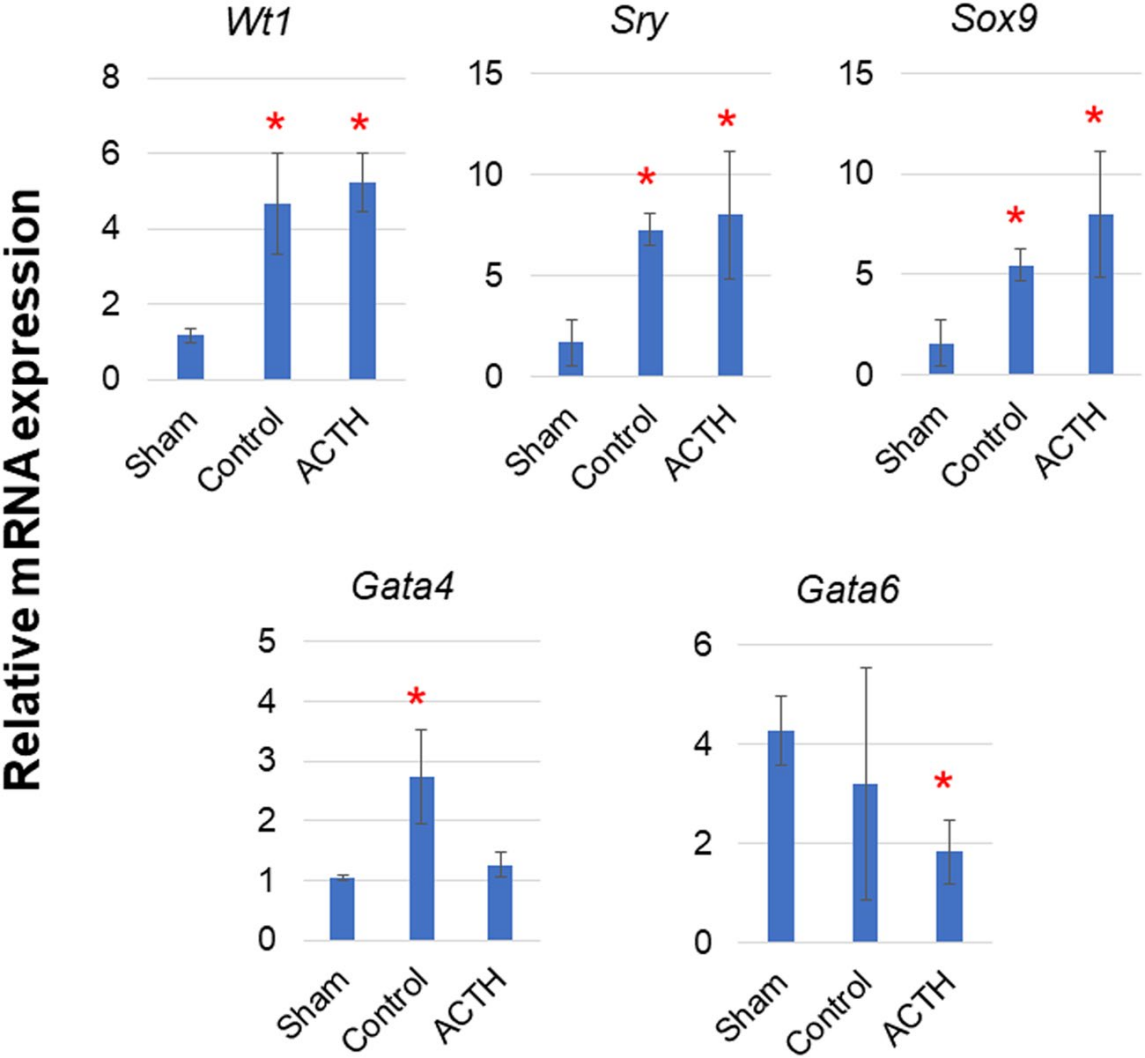
Relative expression of transcriptional regulators in adrenal tissues. Rats with Sham operation (Sham), adrenocortical autotransplantation (Control group) or adrenocortical autotransplantation plus ACTH (ACTH group) were sacrificed at POD14. Relative expression levels in the adrenal tissues were evaluated by RT-qPCR. Changes in transcription level were analysed by ANOVA with a Steel multiple comparisons test ^*^P < 0.05 vs. Sham rats. Wt1: Wilms tumour 1; Sry: Sex-determining region Y; Sox9: SRY-box 9; Gata: Gata-binding factor.

**Figure 4:**
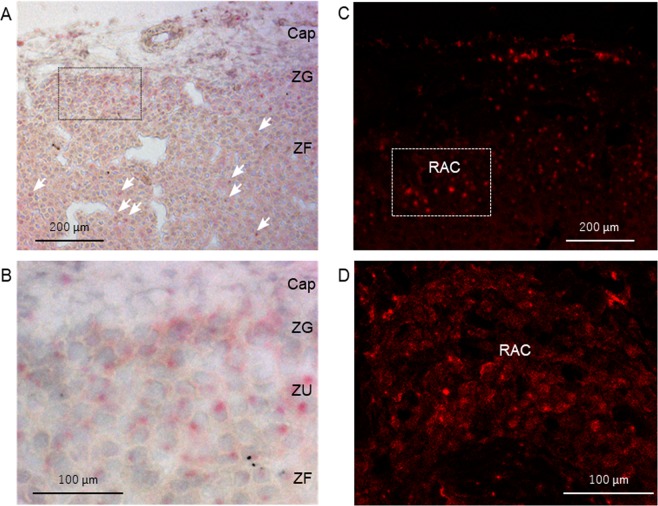
RNAscope in adrenal gland with sham. *Sry* expression was detected in the adrenal layers especially ZU and ZG. (**A**) 004Cow-power field; (**B**) High-power field. *In situ* hybridisation with RNAscope in the adrenocortical autograft at POD14. *Sry* expression persisted in the autograft at POD14. (**C**) Low-power field, (**D**) High-power field. Cap: capsule; ZG: zona glomerulosa; ZF: zona fasciculate; ZU: undifferentiated zone; RAC: renewal adrenocortical cells.

**Figure 5:**
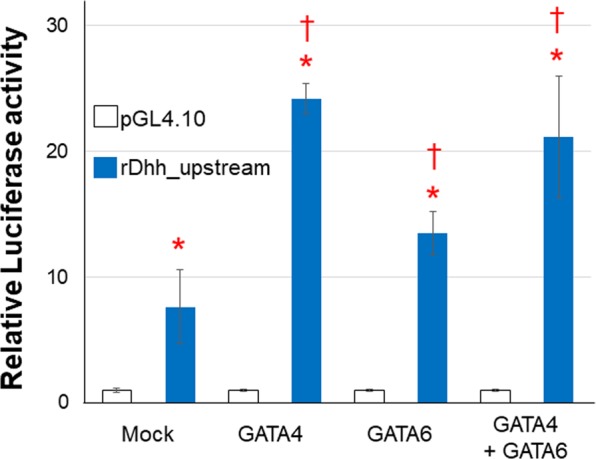
Luciferase activity in the rat *Dhh* upstream region of H295R cells. Four days after transfection, cells were lysed and luciferase activity was measured. Statistical comparisons between the pGL4.10 (pGL4.10) and the Dhh_−2000/+50-pGL4.10 (*Dhh*_upstream) were performed by Student’s *t* test; *P < 0.05 vs. the pGL4.10. Multiple comparisons of luciferase activity between the pCMV6-Entry (Mock) and the GATA4, GATA6 and GATA4 + GATA6 were performed using ANOVA with a Bonferroni correction; ^†^*P* < 0.05 vs. Mock. Dhh, Desert Hedgehog; Gata: Gata-binding factor. N = 6 in each condition.

*Wt1* was upregulated in the control- (4.0-fold) and ACTH-stimulated (4.5-fold) adrenal autografts compared with that in the sham adrenal gland (Fig. 3). We were, then, the first to identify *Sry* and *Sox9* expression in adult adrenal cortex using their cDNA in qRT-PCR. Both were elevated in autografts independently of ACTH stimulation (*Sry*: 4.3-fold change, *Sox9*: 3.4-fold change) (Fig. 3). Cycle sequence analysis disclosed that these PCR products were indeed *Sry* and *Sox9*. RNAscope analysis also confirmed that *Sry* and *Sox9* were localised in the adrenal gland and the autograft. *Sry* and *Sox9* were detected in the zona glomerulosa (ZG) and the estimated undifferentiated zone (ZU), respectively (Fig. 4A,B; Supplementary Fig. 1). *Sry* was detected at low levels in the capsule and the zona fasciculate (ZF). In the adrenocortical autograft, *Sry* was expressed in the stromal cells adjacent to the remnant adrenocortical cells. It was also found in the remnant adrenocortical cells around the capillary circumference at POD14. GATA4 and GATA6 are *Shh* transcriptional regulators in the limb bud^15^. *Gata4* showed a 2.6-fold increase in the autograft (Fig. 3). In the ACTH-stimulated autograft, the transcription factors remained upregulated but both *Gata4* (0.5-fold change) and *Gata6* (0.6-fold change) were inhibited from linking to *Dhh* compared with that in the control autografts (*Gata4*: 0.5-fold change, *Gata6*: 0.6-fold change) (Fig. 3). Therefore, *Gata4* and/or *Gata6* were considered *Dhh* regulators in the adrenal autografts. To clarify this hypothesis, a luciferase reporter assay was conducted on the proximal upstream region of the rat *Dhh* gene including the GATA binding motif at −226/−220. Co-transfection of the rDhh-2000/+50 region (Dhh_−2000/+50_pGL4.10) with the pCMV6-Entry vector significantly increased luciferase activity by ~8× in the H295R cells, human Adrenal gland carcinoma cell line (Fig. 5). Therefore, there may be transcriptional activity in the *Dhh* upstream region even in adrenocortical cells. The rDhh promoter was significantly upregulated by co-transfection with *Gata4* and/or *Gata6* in H295R cells (Fig. 5). GATA binding motifs are conserved in the upstream region of mouse *Dhh* (−230/−224) and human *DHH* (−1024/−1027, −1031/−1034, −1075/−1081). For this reason, transcriptional regulation of adrenal *Dhh* expression by GATA4 and GATA6 might be important in both species. There is a TATA box-like sequence at −517/−514 and typical CCAAT boxes at −610/−606 and −758/−754. Nevertheless, *in silico* analysis suggested that they do not initiate *Dhh* transcription at their distance and that a different transcription initiation site may be present. The typical GC boxes at −49/−44, −27/−22 and −19/−15 were deemed potential promoter region sites. The GATA binding motif could affect this GC boxes activities in H295R cells (Supplementary Fig. 2).

### Effect of ACTH on adrenocortical regeneration

Foetal adrenocortical cells are derived from *Gli1*-expressing cells^16^. We examined the effects of ACTH on the factors determining adrenal development and steroidogenesis in autografts wherein ACTH repressed *Gli1*.

**Figure 6:**
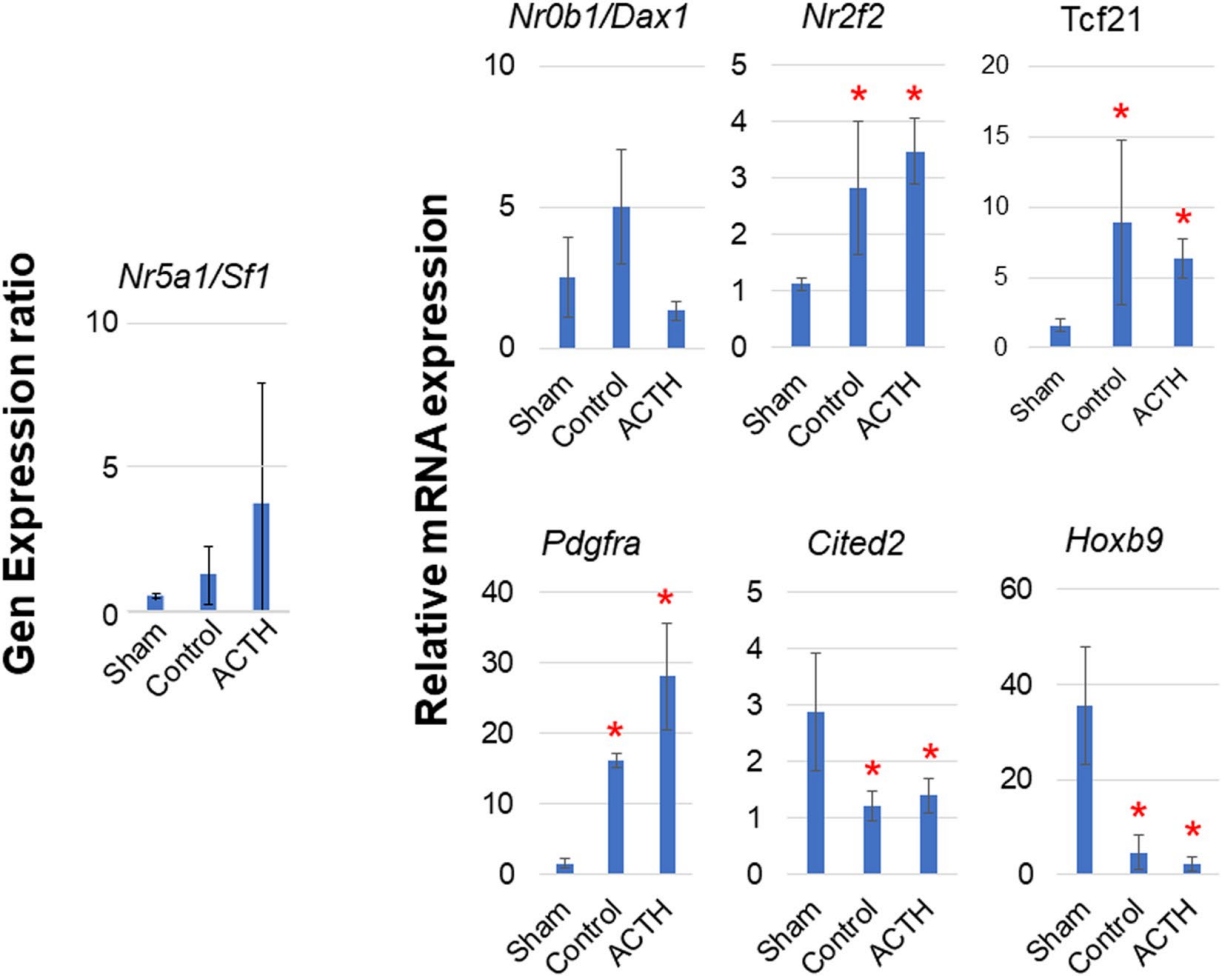
Relative expression of regeneration factors in adrenal tissues. Rats with Sham operation (Sham), adrenocortical autotransplantation (Control group) or adrenocortical autotransplantation plus ACTH (ACTH group) were sacrificed at POD14. Relative expression levels in the adrenal tissues were evaluated by RT-qPCR. Changes in transcription level were analysed by ANOVA with a Steel multiple comparisons test; ^*^P < 0.05 vs. Sham rats. Nr5a1/Sf1: nuclear receptor subfamily 5 group A member 1/steroidogenic factor 1; Nr0b1/Dax1: nuclear receptor subfamily 0 group B member 1/dosage-sensitive sex reversal, adrenal hypoplasia critical region, on chromosome X, gene 1; Hoxb9: homeobox B9 Nr2f2: nuclear receptor subfamily 2 group f member 2; Tcf21: transcription factor 21; Pdgfra: platelet-derived growth factor receptor alpha; Cited2: Cbp/p300 interacting transactivator with Glu/Asp-rich carboxy terminal domain 2.

In ACTH-stimulated autografts, *Nr5a1*/*Sf1* and *Dax1* were not significantly upregulated relative to that in the sham group (Fig. 6). However, ACTH stimulation did not change the level of the stromal markers *Nr2f2*, *Tcf21* and *Pdgfra* compared with that in the control autografts (Fig. 6). *Dhh* and *Gli1* might be involved in adrenocortical cell differentiation and alter the levels of *Nr5a1/Sf1* and *Dax1* in adrenocortical autograft cells^8^. In contrast, the capsule cells could be under the control of other regulators such as *WT1*. Transient ACTH stimulation had a conflicting effect on the regenerations of autograft capsule and adrenocortical cells.

CITED2 co-ordinately controls *Nr5a1*/*Sf1* mRNA accumulation in the adrenogenital primordium (AGP) along with *WT1*^17^. We found that *Cited2* was not upregulated in the autograft (0.4-fold change) (Fig. 6). Therefore, CITED2 might not participate in *Nr5a1*/*Sf1* regulation there. *Hoxb9* is an adrenal steroidogenic cell marker in the AGP^18^. In the autograft, *Hoxb9* was also downregulated (0.1-fold change) (Fig. 6). For this reason, adrenal steroidogenic-like cells in the AGP might be absent in the autografts at POD14. In other words, *Nr5a1*/*Sf1* found in the autografts at POD14 might be expressed in not newly generated adrenocortical cells, but in survival adrenocortical cells.

### Adrenocortical autograft as a bipotential gonad?

Gonads and adrenal glands that originated from an AGP appeared in the form of a coelomic epithelium between the urogenital ridge and the dorsal mesentery. AGP can be detected at embryonic day (E) 9.0 in mice^19^. Adrenal- and gonad anlages progressively individualise from E9.5 to E10.5 and are distinct by E13. Primordial germ cells reach the sexually undetermined gonadal anlage stage by E10. After E11.5-E12, the bipotent gonad differentiates into the testis or ovary with or without *Sry* and by *Sox9* upregulation or downregulation.

**Figure 7:**
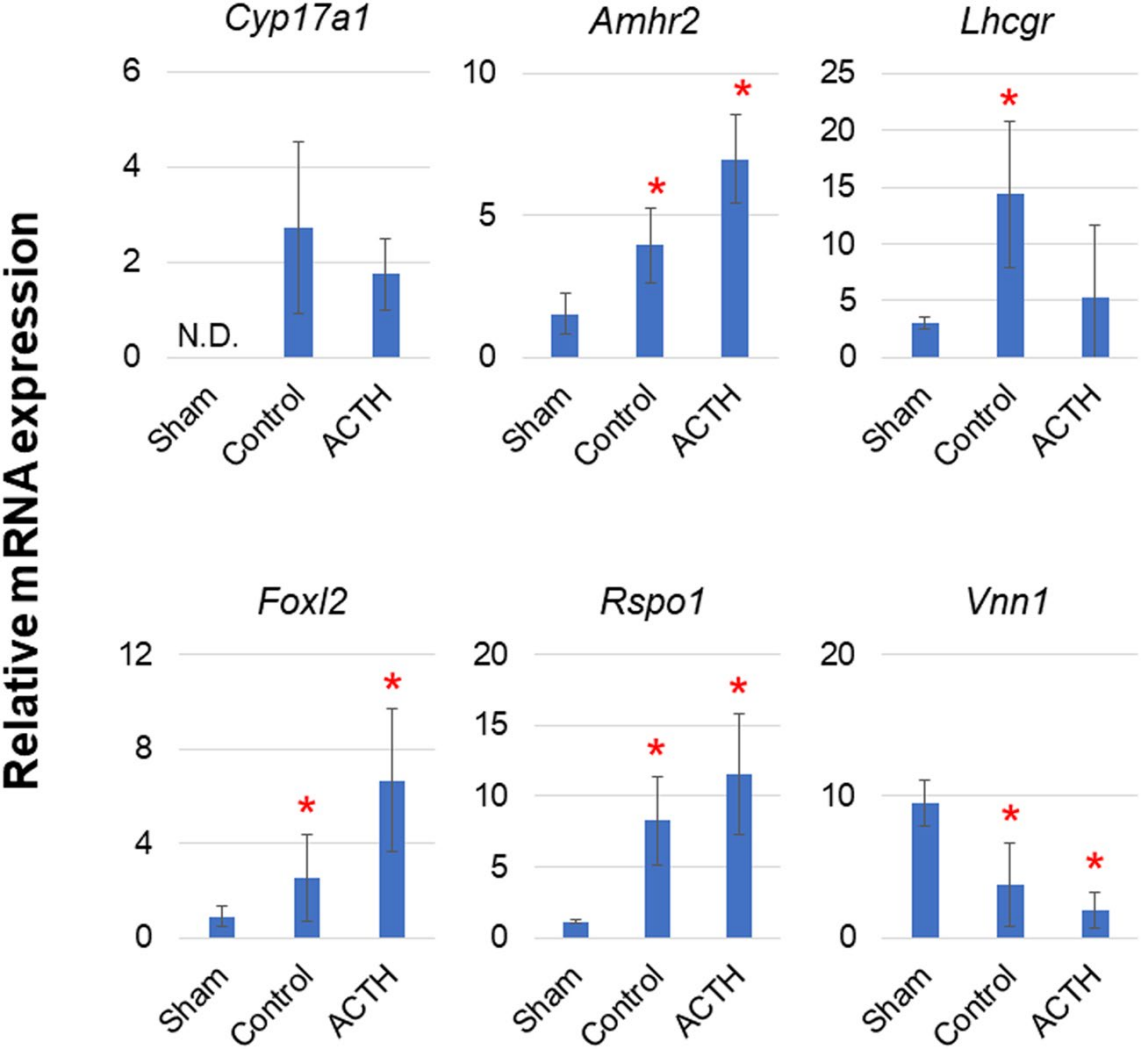
Relative expression of gonadal markers in adrenal tissues. Rats with Sham operation (Sham), adrenocortical autotransplantation (Control group) or adrenocortical autotransplantation plus ACTH (ACTH group) were sacrificed at POD14. Relative expression levels in adrenal tissues were evaluated by RT-qPCR. Changes in transcription level were analysed by ANOVA with a Steel multiple comparisons test ^*^P < 0.05 vs. Sham rats. Cyp17a1: cytochrome P450 family 17 subfamily A member 1; Amhr2: anti-Mullerian hormone type 2 receptor; Lhcgr: luteinising hormone/choriogonadotropin receptor; Foxl2: forkhead box L2; Rspo1: R-spondin 1; Vnn1: vanin 1.

We used gonadal marker detection to investigate whether gonadal differentiation occurs in adrenocortical autografts. Normally, rodent *Cyp17a1* is suppressed in the adrenal gland by a DNA methylation mechanism but is expressed in the gonad and placenta^20^. Even in the present study, we found no *Cyp17a1* expression in the adrenal glands of sham-operated rats. However, the adrenocortical autografts at POD14 presented with relatively upregulated *Cyp17a1* (Fig. 7). Autografts might show changes in their DNA methylation patterns only, but remain adrenal glands with remaining adrenocortical cells, without differentiating to gonadal tissues. We then examined other gonadal markers such as AMHR2 which is specific to AMH target tissues^21^. With or without ACTH stimulation, *Amhr2* was upregulated in a 2.6-fold change in adrenal autografts relative to sham rats (Fig. 7). *Amhr2* is expressed in the developing gonads and Mullerian ducts where it mediates AMH-induced regression^22,23^. In the gonad, AMH signalling promotes masculinisation by suppressing ovary-associated processes such as germ cell meiosis and aromatase and *Lhcgr* expression^24^. In the present study, *Lhcgr* showed a 4.8-fold upregulation in the autografts (Fig. 7) even though the gonad was intact in the adrenocortical autografted rat, unlike the *Lhcgr* upregulation found in post-gonadectomy-induced adrenal hyperplasia. Our histological analysis showed no obvious adrenal hyperplasia in the autografts^8^.

FOXL2 is a differentiation factor in the ovary and represses male-specific genes such as *Sox9* there^25^. *Foxl2* showed a 2.8-fold increase in the present study even though the male determinant factor *Sox9* was elevated in the autografts (Fig. 7). *Foxl2* is first detected by the end of the sex determination period^26^. Therefore, undifferentiated gonads occurred in the autografts. The ovarian determiner RSPO1^27^ was also upregulated in the autografts (7.1-fold change) (Fig. 7). Here, feminisation occurred even in the presence of the male adrenal gland. On the other hand, *Vnn1*, a marker for steroidogenic Sertoli, Leydig and adrenocortical cells^28,29^ and a protectant against oxidative stress^30^, was downregulated in the autografts (0.4-fold decrease) (Fig. 7). Undifferentiated bipotential gonad-like tissues with few or no steroidogenic cells were found in the adrenocortical autograft at POD14. Therefore, gonadal primordia with bipotential testicular and ovarian functions^31^ could be present in autografts.

## Conclusion

This report is the first to clearly demonstrate GATA4 and GATA6 were transcription factors in the regulation of *Dhh* expression in rat adrenocortical autografts. In the present study, only male rats were used and further examinations using female rats are needed to assess the difference of sex. Additionally, transient ACTH stimulation is not considered effective for maintaining the level of *Dhh* in autografts. Thus, future experiments with the long-term ACTH stimulation for autograft need to be performed.

## Methods

### Animals

Twenty-six male Wistar rats (age 7 wks, weight 210–270 g) were purchased from Shimizu Laboratory Supplies (Kyoto, Japan) and housed in a sound-attenuated, light-controlled room (12 h light-dark cycle: lights on at 8:00 and off at 20:00; constant 25 ± 1 °C and 50 ± 10% relative humidity) for 2 wks before the operation. Food and water were provided ad libitum. All animal experiments were approved by the Ethics Committee on Animal Experiments at Kansai Medical University (Approval No. 17–051) and conducted in accordance with the Guide for the Care and Use of Laboratory Animals of the Institute for Laboratory Animal Research.

### Effects of 2 hours ACTH stimulation on adrenal glands

Four male Wistar rats (age 9 wks, weight 210–240 g) received subcutaneous injections of tetracosactide acetate (ACTH 1–24; 20 μg/200 g BW) dissolved in 0.9% w/v saline (the ACTH group). Four other rats received subcutaneous injections of 0.9% w/v saline (the saline group) between 8:00 and 9:00 were decapitated after 2 h. Four age-sex matched rats were used as the intact group. Between 10:00 and 11:00, their adrenal glands were rapidly excised and stored at −80 °C until RNA isolation.

### Adrenocortical autotransplantation

Adrenocortical autotransplantation was performed in twelve 9-wk-old rats as previously described^32^. Briefly, bilateral adrenal glands were resected and divided into four pieces and the medullae were discarded. Adrenocortical autografts were autotransplanted with a pair of fine scissors into two abdominal muscle pockets or the right biceps femoris. Sham operations without adrenalectomy were performed simultaneously. Animals were maintained on saline without any glucocorticoid replacement following the adrenalectomy^33^. ACTH stimulation was performed by injecting tetracosactide acetate (20 μg/200 g BW) dissolved in 0.9% w/v saline 2 h before adrenocortical autotransplantation into abdominal muscle pockets. One rat with ACTH stimulation died within 2 wks after surgery. Two wks after surgery, the adrenal tissues were rapidly excised for gene expression and histological analyses.

### Reverse transcription and quantitative (RT-q) PCR

Total RNA was isolated from each adrenal tissue sample with Sepasol-RNA I Super G reagent (Nacalai Tesque, Kyoto, Japan). Single-strand cDNA was synthesised with a PrimeScript RT reagent kit and gDNA Eraser (TaKaRa Bio Inc., Kyoto, Japan). The mRNA expression levels were determined by quantitative PCR on a Roter-Gene Q platform (Qiagen, Venlo, The Netherlands) using Thunderbird qPCR Mix (Toyobo, Fukui, Japan) and the gene-specific primers listed in Table 1. To test the amplification efficiencies for primer pairs, a 1:10 dilution was used to create a serial dilution series with the undiluted rat adrenal gland or testis cDNA as a starting point. We calculated the amplification efficiency for each primer pair (Table 1). Relative target gene expression levels were evaluated by the 2^−ΔΔCt^ method^34^ using *Hprt1* as an internal control according to previous our studies^8,32^. The 2^−ΔΔCt^ method assumes that primer amplification efficiencies are similar (usually between 90–110%) among target genes and the internal control. All primers efficiencies except those for Angpt2 and Nr5a1/Sf1 ranged from 90 to 110%. We therefore applied the Pfaffl method^35^, which can account for any differences in efficiency, for Angpt2 and Nr5a1/Sf1 to confirm their reproducibility.

**Table 1:** Rat Primers for quantitative RT-PCR.

Name		Forward (5′ to 3′)	Reverse (5′ to 3′)	Amplification efficiency (%)
*Vegfa*	2303F-2398R	aaacacgacaaacccatccc	aaaaacgtctggtcggaacc	95.6
*Angpt1*	1525F-1611R	ggcaaacagagcagcttgatc	aagggcgcatttgcacatac	90.7
Angpt2	337F-427R	acaacacacagtggctgatg	ttctgcaccacattctgctg	87.8
*Shh*	831F-980R	ctgggtctactatgaatccaaagctc	ccgggacttagatccttcactaac	91.5
*Dhh*	1507F, 1637R	accaggctttgcaagaaacc	ctcagattgcctaaaccacagc	92.3
*Gli1*	2080F-2191R	gttgctatggatactagagggctac	catatcccagagtgtcagcagaag	104.8
*Disp1*	1188F-1288R	ccaactatccgtataagtatgcagaagag	ccagtctacttctcttctctctctctc	103.8
*Wt1*	2119F-2203R	gtatatcttcagagatctactttcctcctc	gtacactctaaagacaccccagatg	95.6
*Sry*	235F-320R	agggttaaagtgccacagagg	ttgttgtcccattgcagcag	97.7
*Sox9*	3566F-3686R	tgaacaacgcaagcttctgc	aatccgtacactctccaaccac	97.7
*Gata4*	2458F-2591R	gtttaggtgaggagaaggcacatc	ctctagttttacagagggtaggagatg	94.3
*Gata6*	1663F-1798R	cctcctcctctaattcagatgactg	gaatacttgaggtcactgttctcagg	90.7
*Nr5a1/Sf1*	690F-837R	ctacctctatcctgccttctctaacc	cagctgcaatatgagctctggtac	82.7
*Dax1*	1214F-1292R	gagagtcttcagtggagaactcag	ccgatctgatctggtactctctttg	91.5
*Cited2*	1314F-1398R	cacctcccttatgtagttgaaagtatctag	gggagaaagtgaggaaacaaggag	91.5
*Hoxb9*	768F-871R	ctccccagctcactcttactatttatg	gaggggctttaaagaggagatacc	93.5
*Nr2f2*	1134F-1243R	ccatagtcctgttcacctcagatg	gtactggctcctaacgtactcttc	90.4
*Tcf21*	24F-173R	ggggagataaagctctagtttccc	gaaagggtctctaaagtacggagttg	95.6
*Pdgfra*	4140F-4272R	cccttactaagtagatgacgagtttgg	cactacttacactctgctctctaggg	100
*Cyp17a1*	1592F-1663R	ccacagtacaatcttagaggtgctag	ctagaaaatggggtaggaggaaggag	97.3
*Amhr2*	1737F-1815R	ctctaagtcctgagcctgtaagtg	gtcagcctgtacagagttcatatgag	91.1
*Lhcgr*	215F-332R	ctatctctcacctatctccctgtcaaag	cattagcttctatcctttccagggaatc	90.4
*Foxl2*	1856FF-1966R	cacctccagaccaggtctttatatatatac	ctccgatgaatgttttattctctccttttc	101.4
*Rspo1*	1370F, 1455R	cactcttgaggtcacagaagatatttcc	ctctcagttacgccttctaagagc	93.5
*Vnn1*	900F-973R	ctataggcatgggagtcaatttcctag	gataccacttcctgtcattctcctc	99.5
*Hprt1*	754F, 890R	cctgttgatgtggccagtaaag	atcaaaagggacgcagcaac	95.6

### RNAscope

Adrenal glands and autografts were fixed by immersion in 4% formaldehyde with 0.1 M phosphate buffer (pH 7.4) at 4 °C overnight. The tissues were immersed in 30% w/v sucrose solution for cryoprotection. Fixed, frozen tissues were embedded in optimal cutting temperature compound, cut into 10-µm-thick sections and mounted on Superfrost Plus slides (Thermo Fisher Scientific, Waltham, MA, USA). The sections were air-dried at −20 °C and stored at −80 °C until use. When required, they were returned to 20–25 °C, washed once with distilled water and baked at 60 °C for 30 min. *In situ* hybridisation was conducted using the RNAscope 2.5HD Reagent Kit (Singleplex, RED; ACD LLC, Santa Ana, CA, USA), Probe-Rn-Sry (ACD LLC, Santa Ana, CA, USA), Probe-Rn-Sox9 (ACD LLC, Santa Ana, CA, USA) and Positive Control Probe-Rn-Polr2a (ACD LLC, Santa Ana, CA, USA) or Negative Control Probe-DapB (ACD LLC, Santa Ana, CA, USA) according to the manufacturer’s protocol. After the final amplification, fast-red chromogenic detection was performed. The slides were treated with DAPI Fluoromount-G (SouthernBiotech, Birmingham, AL, USA) and sealed with coverslips. The *Sry* and *Sox9* signals were visualised under a confocal laser microscope (LMS700; Carl Zeiss AG, Oberkochen, Germany). Bright-field images of the fast-red staining were captured with an Eclipse E-1000M digital camera (Nikon, Tokyo, Japan).

### Expression vector and reporter plasmid

The reporter plasmids pGL4.10[luc2] and pGL4.74[hRluc/TK] were purchased from Promega (Madison, WI, USA). The pGL4.74[hRluc/TK] which encodes *Renilla* luciferase was used as an internal control for transfection efficiency. The 2,000-bp upstream region and the 50-bp downstream region of the *Dhh* transcription start site^10^ were amplified with KOD Fx, rDhh_-2000F_SacI (5′-TCCGAGCTCctgagcaagccatgaggagca-3′; the SacI site is underlined) and rDhh_+50R_BglII (5′-GAAGATCTggtttctgctgcccagctccgg-3′; the BglII site is underlined). The PCR products were cloned into pGL4.10[luc2] (Dhh_−2000/+50_pGL4.10).

The expression, pCMV6-Entry, rGATA4-pCMV6 and rGATA6-pCMV6 vectors were purchased from Origen (Rockville, MD, USA). Rat *Gata4* and *Gata*6 were subcloned into pCMV6-Entry (rGATA4-pCMV6) and pCMV6-Entry (rGATA6-pCMV6), respectively.

All constructs were confirmed to have no mutation, no insertion, and no deletion by sequencing analysis with a BigDye Terminator Cycle Sequencing Reaction Kit (Applied Biosystems, Foster City, CA, USA) and an ABI PRISM 3100 Genetic Analyzer (Applied Biosystems, Foster City, CA, USA). Both strands were read with sequence primers.

### Luciferase reporter assay

H295R cells (human adrenal carcinoma) were grown in DMEM:F12 medium (Thermo Fisher Scientific, Tokyo, Japan) supplemented with 6.25 ng/mL each of insulin, transferrin and selenium, 1.25 mg/mL bovine serum albumin (BSA) and 5.35 ng/mL linoleic acid at 37 °C and 5% CO_2_. The final concentration was adjusted to 2.5% with Nu-Serum I (Corning Inc., Corning, NY, USA).

Cells were seeded in 24-well cell culture plates at a density of 2.5 × 10^5^/well. Two types of luciferase plasmids and one expression vector were co-transfected with Lipofectamine^TM^ 3000 Transfection Reagent (Thermo Fisher Scientific, Tokyo, Japan) according to the manufacturer’s protocol. The following amounts of co-transfected plasmids and vectors were placed in each well: 200 ng firefly luciferase-encoding reporter plasmid (pGL4.10), 20 ng Renilla luciferase-encoding internal control plasmid (pGL4.74) and 200 ng expression vectors (pCMV6-Entry, rGata4-pCMV6, or rGata6-pCMV6). Approximately 96 h after transfection, luciferase activity was sequentially measured in duplicate using a PicaGene Dual Sea Pansy Luminescence Kit (TOYO INK CO. Ltd., Chuo-ku, Tokyo, Japan) and the 2030ARVO X multilabel reader (PerkinElmer Japan Co. Ltd., Yokohama, Japan) according to the manufacturer’s protocol. Briefly, 20 µL cell lysate per well in passive lysis buffer was transferred to an OptiPlate-96 plate (SUMILON, Tokyo, Japan). *Firefly* luciferase luminescence (FLU) from the pGL4.10 plasmids and *Renilla* luciferase luminescence (RLU) from the pGL4.74 plasmid were measured independently. Relative luciferase activity per well was calculated by dividing FLU by RLU. Relative luciferase activity was standardised by the corresponding control conditions, namely, either co-transfection of a pGL4.10 plasmid with a pCMV6-Entry, rGATA4-pCMV6, rGATA6-pCMV6 or rGATA4-pCMV6 with rGATA6-pCMV6 vectors. Activity levels were expressed as the mean of ≥6 independent experiments ± standard error (SE).

### Statistical analysis

Normal distribution was analysed by the Shapiro-Wilk normality test. ANOVA with a Steel multiple comparisons test was used for the normally distributed factors. For *Shh* and *Dhh* data, which were not normally distributed, the Mann-Whitney *U* test with a Bonferroni correction was used. Comparisons between *pGL4* and *Dhh* were performed by Student’s *t* test. Multiple comparisons between the Mock-Dhh and the other Dhh were performed using ANOVA with a Bonferroni correction. Statistical analyses were performed in IBM SPSS Statistics v. 21.0 (IBM Corp., Armonk, NY, USA). All values were two-sided with statistical significance set at 0.05.

## Supplementary information

Supplementary Figure1,2.
