# Supplementary material for: GATA4/6 regulate DHH transcription in rat adrenocortical autografts
Source: Sci Rep. 2020 Jan 16;10:446. doi: 10.1038/s41598-019-57351-5 (PMC6965091; doi:10.1038/s41598-019-57351-5)
Supplement: Supplementary file 1 — Supplementary Figure1,2. [file 41598_2019_57351_MOESM1_ESM.pdf]

GATA4/6 regulate DHH transcription in rat adrenocortical autografts

Takashi Yoshida[1,2], Nae Takizawa[1,2], Tadashi Matsuda[2], Hisao Yamada[1], Masaaki Kitada [1] and Susumu Tanaka[1]*

[1]**Department of Anatomy**, Kansai Medical University, Hirakata, Osaka 573-1010, Japan
[2] Department of Urology and Andrology, Kansai Medical University, Hirakata, Osaka 573-1010, Japan

*Correspondence to: Dr Susumu Tanaka,
Department of Anatomy,
Kansai Medical University,
2-5-1 Shin-machi, Hirakata, Osaka 573-1010, Japan
Phone: +81-72-804-2304
Fax: +81-72-804-2039
E-mail: tanakass@hirakata.kmu.ac.jp

S.Figure1

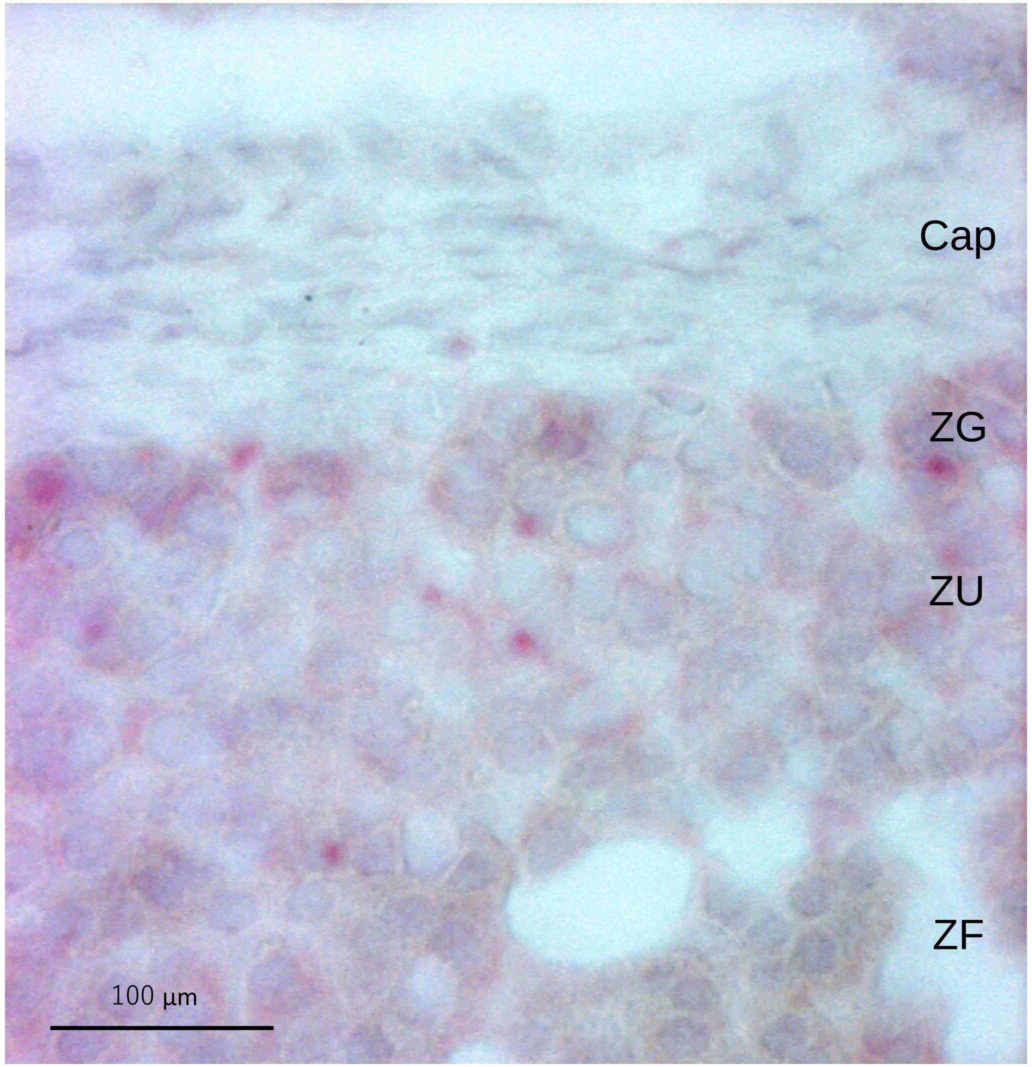

Supplementary Fig. 1. Sox9 expression in adrenal gland.

Sox9 expression with *in situ* hybridisation using RNAscope. *Sox9* was detected in ZG in the sham operated-rat. Sox9: SRY-box 9.

Cap: capsule; ZG: zona glomerulosa; ZF: zona fasciculate; ZU: undifferentiated zone; RAC: renewal adrenocortical cells

S.Figure2

-758 -754

AGGCCAAAGGAGAAAACACTCAAGGACATCCTTCTCTGTGAAGTGAGTTTGAGA**CCAAT**CCT

CAAT box

GAACTACATTTGATCCTGTCAAAAGAGGAGAGGGGAATAAAAAGTGACCCTCGAGTATCTG

GCAACCATTTGAGAGGACACCAACCTTGGAAAGTCCTGGTTTGGTAGATCAGCAGGTAACAT

-610 -606

CACTTAACGCACATGC**CCAAT**TAACGTCCCACTGTGGAAGAAGAAAGCCGACTCTAAAAAGC

CAAT box

-517 -514

CCTCTGACCTCCACAGGTGTGCTATGGCGTCCTGCACACGCACAACA**TATA**CAGACACAGCA

TATA box

GTGACAAAAAAATATGTCCCAAACTGGATTGTGAGTGTCAGGCTCTGTTCCCAAGAAGACAT

AAAACCACCAAGGAAAATGCTCCTCTGGGCGTCAGCCTTGGTTATGGGTGAGTATGAAGAGA

AACCCGGGAGGACTTGAGGAGCACAGAGAAGGGAGGCTTTGATGGTTTGCCCAAAGGGCTTG

GATGGCCTTGGAACTCACACTTCCTGGAGGACAGCAGAGGACATGGTCTGCCAGGGTAGACT

-226 -221

TTAGGAGAGCAAAGACCAGTGCCCAGGAA**GATA**GCTTGGTTCCTAATTTAGACTCGGAGGTA

GATA binding motif

GAAGGATGCTTATCCAGGCAGCCAGGACTCTAAAGCTTCAGGGATTTCCTAAGCAGGTGCCT

GCATGGTTTCCAGGATGGGACACACTTCAATGGAGTAATGGAACAGTGGTGACCGCTAGATC

-49 -44 -27 -15

CCCCGCCCGGGTGGGAGGG**GGCGGG**ACAGCCCTGGGAGAAG**GGCGGG**G**GGCGGG**GCCAGGGA

GC box GC box GC box

+**1**

CCACTTAAGGCGGAGTCAGGGCGTTTCTGGGAGCACCGGAGCTGGGCAGCAGAAACC

Supplementary Fig. 2. Schematic representation of the regulatory region of the rat Dhh gene.
The start of exon 1 of rat Dhh gene is marked as +1 and the exon is highlighted in green. The 6-bp region of the putative GATA binding site is shown in bold and has been highlighted in yellow at -226/-221. The TATA box-like sequence at -517/-514, the typical CCAAT boxes at -610/-606 and -758/-754, and the typical GC boxes at -49/-44, -27/22 and -19/-15 are shown in bold and have been underlined.
